# Dim Light at Night Impairs Daily Variation of Circulating Immune Cells and Renal Immune Homeostasis

**DOI:** 10.3389/fimmu.2020.614960

**Published:** 2021-01-22

**Authors:** Monika Okuliarova, Nikoleta Mazgutova, Miroslava Majzunova, Valentina Sophia Rumanova, Michal Zeman

**Affiliations:** Department of Animal Physiology and Ethology, Faculty of Natural Sciences, Comenius University, Bratislava, Slovakia

**Keywords:** chronodisruption, leukocyte trafficking, chemokines, monocytes, renal redox balance

## Abstract

Dim light at night (dLAN) has become a pervasive part of the modern world, and growing evidence shows its association with increased health risks. Though this link is attributed to a disturbed circadian clock, the underlying mechanisms that can explain how circadian disruption from dLAN causes negative health effects remain unclear. Here, we exposed rats to a light–dark cycle (12:12 h) with low-intensity light at night (~2 lx) for 2 and 5 weeks and explored the steady-state pattern of circulating immune cells and renal immune-related markers, which are well controlled by the circadian clock. After 5 weeks, dLAN impaired the daily variation in several types of white blood cells, especially monocytes and T cells. Two-week dLAN caused a reduction in blood monocytes and altered gene expression of macrophage marker *Cd68* and monocyte-attracting chemokine *Ccl2* in the kidney. Interestingly, dLAN decreased renal 3-nitrotyrosine levels and resulted in up-regulation of the main endogenous antioxidant pathways, indicating a disturbance in the renal redox balance and an activation of compensatory mechanisms. These effects paralleled the altered renal expression of the molecular clock components and increased plasma corticosterone levels. Together, our results show that chronic exposure to dLAN weakened the circadian control of daily variation of circulating immune cells and disturbed renal immune and redox homeostasis. Consequences of this dLAN-disturbed immune balance on the ability of the immune system to cope with other challenges should by clarified in further studies.

## Introduction

Photic information affects the proper functioning of almost all biological systems. This is mainly because light mediates not only visual perception but also provides a non-image-forming stimulus responsible for circadian entrainment (1). Through this process organisms align their endogenous circadian rhythms with the natural 24 h light and dark cycles in order to coordinate the timescale of their physiology and behavior with the cyclic environmental changes (2). In mammals, circadian rhythms are governed in a hierarchical manner by a master clock, which resides in the suprachiasmatic nuclei (SCN) of the hypothalamus and communicates with countless subordinate oscillators located in both the brain and periphery (3, 4), including the cells and tissues of the immune system (5).

On-going progress in lighting technologies and the parallel changes in human lifestyles closely relate to the increasing levels of both indoor and outdoor dim light at night (dLAN), which has caused the active phase to become virtually independent of a solar day (6). In general, dLAN means excessive and inappropriately timed light exposure, e.g., in light-polluted urban areas or bedtime use of light-emitting electronic devices (7). A growing number of epidemiological studies have reported evidence for an association between exposure to dLAN and several pathologies, including obesity (8), breast and prostate cancer (9), increased blood pressure (10), and sleep disorders (11). Because dLAN disturbs the natural light–dark (LD) cycles, circadian clocks are considered the primary system susceptible to improper functioning, underlying the link between dLAN and increased health risks (12, 13). Indeed, after chronic exposure to dLAN (≤ 5 lx), compromised circadian function has been shown experimentally through suppressed night-time melatonin levels (14, 15), attenuated rhythms in locomotor activity (16) and cardiovascular parameters (14), and altered clock gene oscillations (17). Nonetheless, the underlying physiological and molecular mechanisms, how circadian disruption due to dLAN causes detrimental health effects, are still poorly understood.

Immune dysfunction plays an essential role among the mechanisms linking circadian disruption to the development of diseases (18). Various aspects of both innate and adaptive immune functions (e.g., immune cell trafficking, cytokine production and other functional properties of immune cells) display 24-h rhythms and are under circadian control (19). This circadian regulation is maintained both centrally *via* endocrine and autonomic nervous pathways and locally *via* peripheral oscillators present in multiple immunocompetent cells (5). The most prominent circadian oscillations have been recognized for leukocyte trafficking between the bone marrow, blood, and other body tissues, including lymphoid organs (20), the liver, the lung (21), and the kidney (22). Under steady-state conditions in rodents, most of the circulating leukocyte subsets peak at the beginning of the day (the passive phase), whereas maximum exit from circulation occurs during the night (21). These daily rhythms of immune cell trafficking are clearly important to coordinating defense mechanisms (20, 22). For example, time of day dependent presence of aged neutrophils in the renal tissue has been shown to confer protection against fungal infection in mice (22). On the other hand, chronic circadian misalignment induced by experimental jet lag resulted in an activation of immune system-associated pathways in the liver and kidney, steatohepatitis, and inflammatory cell infiltration (23).

Here, we explored a potential circadian-dependent immunomechanism that can link dLAN to negative health consequences. Indeed, recently we reported dLAN-induced health risks by increased hepatic lipid accumulation in dLAN-exposed rats (24). In the current study, we tested the hypothesis that chronic exposure to low-intensity light at night (~2 lx) compromises the circadian system, leading to disturbances in the daily variation of circulating immune cells and leukocyte movement into the renal cortex under homeostatic conditions. This study follows our recently published paper, in which we demonstrated that dLAN resulted in attenuated circadian rhythms in the cardiovascular system and the suppression of nocturnal plasma melatonin levels (14), which represent an important central clock-derived output and a time-giver for peripheral oscillators (25).

## Materials and Methods

### Animals

Male Wistar rats were obtained at the age of 14 weeks from the breeding station of the Institute of Experimental Pharmacology and Toxicology, Slovak Academy of Sciences (Dobrá Voda, Slovak Republic). The animals were housed in plastic cages in groups of three to four rats at an ambient temperature of 21.5 ± 1.3°C and humidity of 55%–65%, and they were provided with a standard pelleted diet and water *ad libitum*. During the acclimation period of four weeks, rats were maintained on a LD cycle of 12:12 h with lights on at 10:00 am, designed as Zeitgeber time 0 (ZT0). At the level of animal cages, room lights emitted white light with an illumination of 150–200 lx and color temperature of 2,900 K.

### Experimental Design

After acclimation, rats (311 ± 28 g) were assigned to either the control group (CTRL, n=15) with the standard lighting regime described above, or to the experimental group (dLAN, n=18), which was exposed to low-illuminance levels of 2 lx during the entire night phase. The dim light conditions were provided by a shaded lamp with LED bulb Star Classic A60 10W (OSRAM GmbH, Germany), which emitted a broad-spectrum white light with a peak of 610 nm and color temperature of 2,700 K. Illuminance and color temperature was measured at the level of cages using an illuminance spectrophotometer CL-500A (Konica Minolta Sensing Europe BV, Germany).

### Blood and Tissue Collection

To examine the numbers and daily pattern of circulating leukocyte populations, rats (CTRL, n=7; dLAN, n=9) were repeatedly blood sampled from the tail vein at ZT9 and ZT21 after 2 and 5 weeks of the experiment. At each time point, sampling was completed within 1 h for all animals. These time points were chosen to encompass night-time melatonin peak, which occurs in the second half of the dark phase in rats (26). Plasma melatonin levels from this experiment have already been reported in our recent paper (14). Rats were immobilized under isoflurane anesthesia, and blood was collected from a lateral tail vein into tubes with either ethylenediaminetetraacetic acid (for immunophenotypic analysis) or heparin (for analysis of the oxidative burst of neutrophils).

After 2 weeks (CTRL, n=7; dLAN, n=8) and 5 weeks (CTRL, n=8; dLAN, n=10) of the experiment, rats were sacrificed under isoflurane anesthesia during the first half of the light phase (between ZT3 and ZT6). Blood was collected into heparin tubes and centrifuged (2,500 g, 10 min, 4°C). The separated plasma was stored at –20°C until performing the corticosterone assay. The left kidney was dissected and cut into three pieces; the middle part was processed for immunofluorescence and both ends were immediately frozen in liquid nitrogen and stored at –76°C until molecular analyses.

### Immunophenotypic Analysis

A suspension of blood leukocytes in phosphate-buffered saline (PBS) was obtained after the lysis of red blood cells using ammonium–chloride–potassium buffer. Thereafter, leukocytes were fixed in 4% phosphate-buffered formaldehyde (Ultra Pure, Polysciences, USA; 87001-890) and washed, and the pellets were diluted to the original blood volume in PBS supplemented with 0.5% bovine serum albumin (BSA) and 0.1% sodium azide (staining buffer). Aliquots of the leukocyte suspension (50 µl) were quadruple or triple stained with a cocktail of antibodies, incubated for 30 min at 4°C in the dark, and analyzed on a BD Accuri C6 cytometer (BD Bioscience, USA). The following fluorochrome-conjugated monoclonal antibodies were used: PE-Cy7 anti-rat CD45 (clone OX-1; Sony Biotechnology, USA; 1611065), FITC anti-rat CD3 (clone G4.18; Thermo Fisher Scientific, USA; 11-0030), APC anti-rat CD4 (clone OX-35; Thermo Fisher Scientific; 17-0040), PE anti-rat CD8a (clone OX-8; Thermo Fisher Scientific; 12-0084), PE anti-rat CD45RA (clone OX-33; Thermo Fisher Scientific; MR6404), APC anti-rat CD161a (clone 10/78; Thermo Fisher Scientific; MR6805), FITC anti-rat granulocytes (clone HIS48; Thermo Fisher Scientific; 11-0570), and PE anti-rat CD43 (clone W3/13; Sony Biotechnology; 1614060). The following rat leukocyte subsets were identified on the basis of their surface markers in the gate for total leukocytes (CD45^+^): T-cells (CD3^+^), helper T cells (CD3^+^CD4^+^), cytotoxic T cells (CD3^+^CD8a^+^), B cells (CD45RA^+^), NK cells (CD3^-^CD161a^+^), neutrophils, and monocytes (HIS48^+^ and side scatter gating). Reciprocal expression of CD43 and HIS48 markers was used to identify classical (CD43^lo^HIS48^hi^) or non-classical monocytes (CD43^hi^HIS48^lo^) (27). The gating strategy for identifying individual populations of peripheral blood leukocytes is given in **Figure S1**. All cytometric data were analyzed using FlowJo software (TreeStar, USA).

### Oxidative Burst of Neutrophils

The oxidative burst of blood neutrophils was evaluated in rats after 5 weeks of dLAN. In this procedure, 50 µl of whole blood was diluted 20 times with PBS containing 20 µM of 2′,7′-dichlorodihydrofluorescein diacetate (H_2_-DCF-DA; MilliporeSigma, USA; D6883) and incubated for 30 min at 37°C in the dark. Thereafter, each sample was divided into two equal parts; one part was stimulated with phorbol-12-myristate-13-acetate (PMA; MilliporeSigma; P8139) at a final concentration of 1 µM and one part served as the unstimulated control (dimethyl sulfoxide was added at a final concentration of 0.1%). After incubation (for 30 min at 37°C in the dark), the cells were washed with PBS and red blood cells were lysed using eBioscience™ RBC Lysis Buffer (Thermo Fisher Scientific; 00-4333-57). After lysis of erythrocytes, the cells were washed, resuspended in 0.3 ml of PBS, and analyzed on a flow cytometer. Within the cell, H_2_-DCF-DA is converted to fluorescent 2′,7′-dichlorofluorescein (DCF), and this fluorescence is a measure of H_2_O_2_ production and is linearly related to the oxidative burst of the stimulated neutrophils (28). The functional activity of the neutrophils was expressed as a fold increase in the median DCF fluorescence of the PMA stimulated samples over the unstimulated samples.

### Immunofluorescence

The kidney samples were washed in cold PBS and fixed in 4% phosphate-buffered formaldehyde overnight at 4°C. Then, the samples were cryoprotected in 1.1 M sucrose, embedded in OCT Cryomount (Histolab Products AB, Sweden; 45830), and stored at –76°C. Frozen kidney sections (8 µm) were prepared on adhesive slides, rehydrated, and incubated with 50 mM NH_4_Cl for 30 min. Thereafter, sections were permeabilized with 0.25% Triton X-100 in PBS for 10 min and blocked with 5% goat serum in PBS for 1 h at room temperature. To identify macrophages, sections were stained with mouse anti-rat CD68 (Bio-Rad, USA; 1:100 dilution; MCA341R) overnight at 4°C. Next, the sections were incubated with an Alexa Fluor 660-conjugated secondary antibody (Thermo Fisher Scientific; 1:750 dilution; A-21055) for 1 h at room temperature. Cell nuclei were counterstained with DAPI (Roche, USA; 1:10,000 dilution; 10236276001). The CD68-positive cells were analyzed using a Zeiss Axioscope (Carl Zeiss, Germany) fluorescence microscope. The cells were counted in 30 randomly selected images of the renal cortex and averaged from 2 sections per individual. The cell numbers were calculated per mm^2^.

### Western Blot Analysis

The samples of the renal cortex were homogenized on ice in 0.05 M Tris-HCl buffer (pH 7.4) supplemented with protease inhibitors. The protein concentrations were determined with the BCA assay kit (MilliporeSigma; 71285). Total proteins (20 µg) were separated with 12% SDS-PAGE and transferred to a nitrocellulose membrane. Membranes were blocked with 5% BSA in Tris-buffered saline containing Tween 20 (TBS-T). Afterwards the membranes were incubated with mouse anti-3-nitrotyrosine antibody (Abcam, 1:1,500 dilution; ab61392) or mouse anti-rat CD68 antibody (Bio-Rad; 1:1000 dilution; MCA341R) overnight at 4°C. Following three washes (3×10 min) with TBS-T, the membranes were incubated with horseradish peroxidase-conjugated secondary antibody (Cell Signaling Technology, USA; 1:2,000 dilution; 7076) for 1 h at room temperature. Both primary and secondary antibodies were diluted in TBS-T containing 1% BSA. All blots were reprobed with mouse anti-glyceraldehyde-3-phosphate dehydrogenase (GAPDH) antibody (clone 6C5; MilliporeSigma; 1:2,000 dilution; MAB374) and incubated for 1 h at room temperature. The signals were visualized with Clarity Western ECL substrate (Bio-Rad; 1705061) using ChemiDoc™ Touch Imaging System (Bio-Rad) and quantified with Image Lab Software. The amount of target proteins was normalized relative to GAPDH and presented in arbitrary units (a.u.).

### Measurement of Superoxide Dismutase Activity

The total superoxide dismutase (SOD) activity was measured in 0.5% homogenates of renal cortex prepared in 0.05 M Tris-HCl buffer (pH 7.4) containing protease inhibitors with the SOD assay kit (MilliporeSigma; 19160-1KT-F), according to the manufacturer’s instructions. The tissue SOD activity was calculated using a SOD standard curve (MilliporeSigma; S7571-15KU) and expressed in units per milligram of proteins (U/mg).

### Corticosterone Assay

Plasma corticosterone levels were determined by a radioimmunoassay (RIA) using the Corticosterone Rat ^125^I RIA kit (DRG Instruments GmBH, Germany; RIA-1364), according to the manufacturer’s instructions.

### RNA Isolation and Real-Time PCR (qPCR)

Tissue samples of the renal cortex were homogenized using a FastPrep instrument (MP Biomedicals, Germany), and total RNA was isolated with an Rneasy Plus Universal Mini kit (Qiagen, Germany; 73404), according to the manufacturer’s instructions. Before reverse transcription, genomic DNA was eliminated by DNase I (Thermo Fisher Scientific, USA; EN0521). For synthesis of complementary DNA, a Maxima First Strand cDNA Synthesis Kit for RT-qPCR (Thermo Fisher Scientific; K1641) was used. The quantity and purity of the isolated RNA were measured with a NanoDrop One spectrophotometer (Thermo Fisher Scientific). The RNA integrity was verified with 1.2% agarose gel electrophoresis. Amplification of cDNA was performed with Maxima SYBR Green qPCR Master Mix (Thermo Fisher Scientific; K0223) and the CFX Connect real-time PCR detection system (Bio-Rad). The relative expression of the target and reference genes was calculated using a standard curve method. The expression of the target genes was normalized to the expression of the ribosomal protein S29 (*Rps29*). Primer sequences are listed in **Table S1**.

### Statistical Analyses

Data are presented as means ± standard error of mean (SEM). Statistical analyses were performed in R (R Core Team, Vienna, Austria). Two-way analysis of variance (ANOVA) with repeated measures and Tukey *post hoc* tests were used to analyze differences in the numbers of individual white blood cell (WBC) subsets between the CTRL and dLAN groups and between two ZT points. For other parameters, CTRL and dLAN groups were compared by the Student’s *t*-test or the Mann–Whitney U test, depending on the normal distribution. A *p*-value of <0.05 was considered to be statistically significant.

## Results

### dLAN Disturbs the Daily Variation and Alters the Number of Circulating Immune Cells

**Figure 1:**
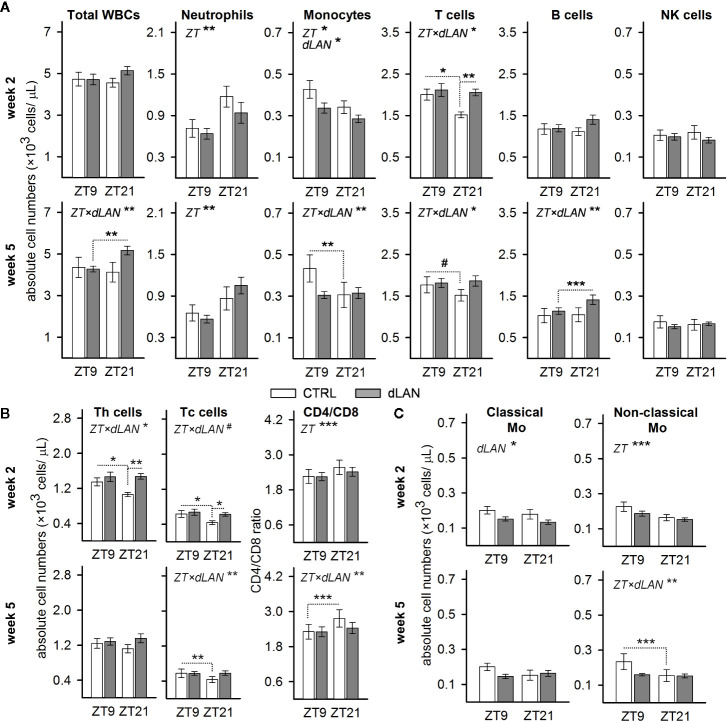
Dim light at night (dLAN) disturbs the daily variation and alters the number of circulating immune cells. **(A–C)** Flow cytometric analysis of peripheral white blood cells (WBCs) collected at ZT9 and ZT21 from rats exposed to either the control light–dark (LD) regime (CTRL) or dLAN (~2 lx) for 2 (upper rows) and 5 (lower rows) weeks. Data represent the mean ± SEM with n = 6–9 per group. **(B)** Numbers of CD4^+^ helper (Th) and CD8^+^ cytotoxic (Tc) T cells and the CD4/CD8 ratio. **(C)** Numbers of classical (CD43^lo^HIS48^hi^) and non-classical (CD43^lo^HIS48^hi^) monocytes. Significant differences were evaluated by two-way repeated ANOVA with the Tukey *post hoc* test for multiple comparisons. Only significant main effects (ZT and dLAN) or interactions (ZT×dLAN) are shown. Dotted lines indicate significant differences between individual groups if an interaction was significant. ^#^ *P* < 0.1, *P < 0.05, **P < 0.01, ***P < 0.001.

In blood, leukocytes exhibit a pronounced daily rhythmicity, which is, for most of the leukocyte types in rats and mice, characterized by a peak at the beginning of the passive (light) phase and a trough at the beginning of the active (dark) phase (29). To test whether dLAN can influence the number of immune cells in blood and their daily oscillations, we evaluated the immunophenotype of circulating WBCs at ZT9 and ZT21 in rats exposed to dLAN (~2 lx) for 2 and 5 weeks. Rats in the control regime had lower numbers of blood monocytes and T cells at ZT21 compared to ZT9, whereas the opposite pattern was observed for neutrophils (**Figure 1A**). After 5 weeks of dLAN, the daily variation was lost or disturbed for most types of WBCs, except neutrophils (**Figure 1A**).

Next, we analyzed whether dLAN affects the absolute number of leukocyte populations in the blood. No differences between the CTRL and dLAN rats were recorded for the total leukocyte count after either 2 or 5 weeks of dLAN (**Figure 1A**). However, 2 weeks of dLAN resulted in a lower number of monocytes and a higher number of T cells, though the increase in T cells was specific for ZT21 (**Figure 1A**). Both CD4^+^ helper and CD8^+^ cytotoxic T cells displayed the same significant changes as the total T cell numbers (**Figure 1B**). Moreover, there were no differences between the CTRL and dLAN rats in the CD4/CD8 ratio, indicating that both T cell subsets responded to dLAN in the same way. A day–night difference was also recognized for the CD4/CD8 ratio, with higher levels at ZT21 compared to ZT9, and this daily variation was impaired in rats exposed to dLAN for 5 weeks (**Figure 1B**).

Because the circulating monocytes are phenotypically and functionally distinguishable into several populations (30), we examined whether the dLAN-induced decrease in total blood monocyte numbers could be attributed to the changes in classical or non-classical monocytes. In rats, reciprocal expression of CD43 and HIS48 can be used to identify classical (CD43^lo^HIS48^hi^) or non-classical (CD43^hi^HIS48^lo^) monocytes (27), which are analogous to murine Ly6C^hi^ and Ly6C^lo^ monocyte subsets (30). After 2 weeks of dLAN, the decline in total monocyte number was mainly due to a decrease in the population of classical CD43^lo^HIS48^hi^ monocytes (**Figure 1C**). On the other hand, the loss of daily variation following 5 weeks of the dLAN regime was mainly explained by the population of non-classical CD43^hi^HIS48^lo^ monocytes, though the pattern was similar in both monocyte subsets (**Figure 1C**).

Collectively, these results indicate that chronic exposure of rats to dLAN can impair daily variation in the main leukocyte populations in the blood.

### *dLAN* Alters Renal Expression of *Cd68* and Monocyte-Attracting Chemokine *Ccl2*

**Figure 2:**
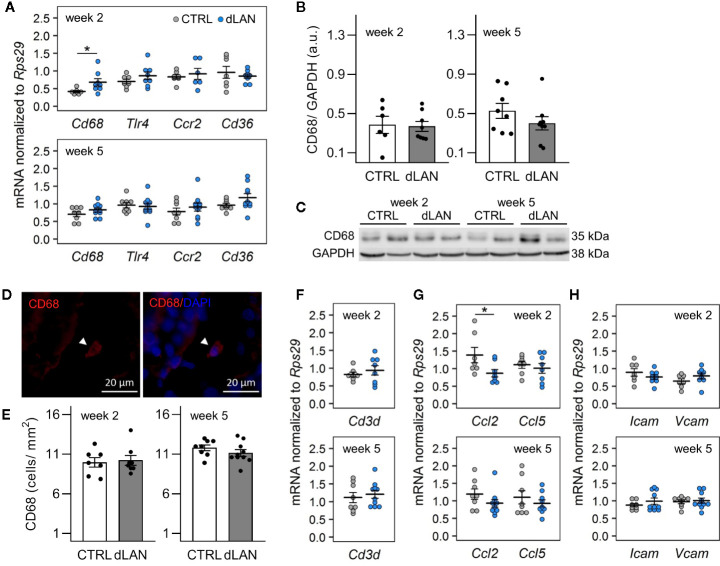
Dim light at night (dLAN) alters renal expression of *Cd68* and monocyte-attracting chemokine *Ccl2*. Rats were exposed to either the control light–dark (LD) regime (CTRL) or dLAN (~2 lx). After 2 and 5 weeks, rats were sacrificed between ZT3 and ZT6 and the kidneys were isolated for qPCR, Western blot and immunofluorescence analyses. **(A, F–H)** Relative mRNA levels of macrophage markers **(A)**, T cell marker *Cd3d* **(F)**, monocyte and lymphocyte chemokines **(G)** and cell adhesion molecules **(H)** for rats exposed to either the CTRL (grey dots, n = 7–8) or dLAN regime (blue dots, n = 7–10) for 2 or 5 weeks. Data are shown as dot plots with the mean (thick horizontal lines) ± SEM (error bars). **(B)** Representative immunoblot of CD68 normalized to GAPDH showing 2 rats per each group. **(C)** Quantification of CD68 from immunoblots. **(D)** Representative immunofluorescent images of macrophage marker CD68 (red) counterstained with DAPI (blue). Arrowheads indicate CD68-positive cells. **(E)** Quantification of CD68-positive cells from immunofluorescent images. Data represent the mean ± SEM with n = 6–10 per group. Black dots indicate the individual data points. CTRL and dLAN groups were compared with the Student’s *t*-test. *P < 0.05.

Based on the dLAN-induced changes in the immune cell numbers in the circulation, we expected the effects on the recruitment of immune cells into tissues. Kidney-resident and infiltrating immune cells are increasingly recognized to play a central role in tissue homeostasis as well as in the modulation of many pathophysiological conditions (31, 32), and recent data show that renal immune pathways can be activated by the disturbed circadian clock (23). Therefore, we quantified specific markers for macrophages and T cells, including chemokines and cell adhesion molecules, in the renal cortex of rats exposed to dLAN for 2 and 5 weeks. Following 2 weeks of the dLAN regime, the rats showed higher renal mRNA levels of a common macrophage marker, *Cd68*, compared to controls (**Figure 2A**), though no differences between groups were detected at the protein level analyzed by Western blot (**Figures 2B, C**) and in the CD68+ cell counts analyzed by immunofluorescence staining (**Figures 2D, E**) after both 2 and 5 weeks of dLAN. No differences between dLAN and CTRL groups were found in the gene expression of toll-like receptor 4 (*Tlr4*), C-C chemokine receptor type 2 (*Ccr2*) and the macrophage scavenger receptor (*Cd36*) (**Figure 2A**). Moreover, renal mRNA levels of a specific marker for T cells, *Cd3d*, were not affected by dLAN (**Figure 2F**).

Next, we analyzed the renal expression of chemokines and cell adhesion molecules, which mediate monocyte and lymphocyte trafficking into tissues. In both steady-state and disease conditions, the important monocyte-attracting chemokine is C-C motif chemokine ligand 2 (CCL2) (33, 34). We found that rats exposed to dLAN for 2 weeks displayed lower *Ccl2* mRNA levels in the kidney compared to controls (**Figure 2G**). dLAN did not affect the gene expression of C-C motif chemokine ligand 5 (*Ccl5*), intercellular cell adhesion molecule-1 (*Icam1*), and vascular cell adhesion molecule-1 (*Vcam1*) (**Figures 2G, H**).

### dLAN Disturbs the Redox Balance in the Kidney

**Figure 3:**
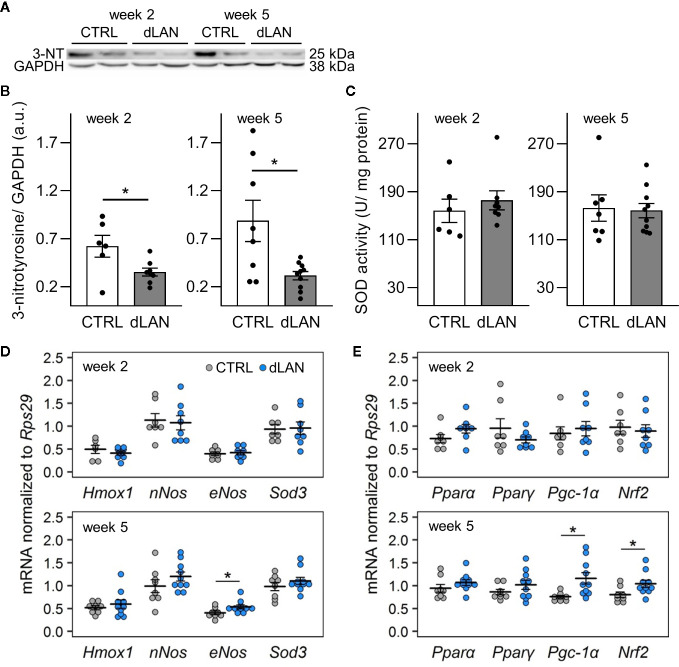
Dim light at night (dLAN) disturbs the redox balance in the kidney. Rats were exposed to either the control light–dark (LD) regime (CTRL) or dLAN (~2 lx) for 2 and 5 weeks. **(A)** Representative immunoblot of renal 3-nitrotyrosine (3-NT) normalized to GAPDH showing 2 rats per each group. **(B)** Quantification of 3-NT from immunoblots. **(C)** Total activity of superoxide dismutase (SOD) in homogenates of the renal cortex. Data represent the mean ± SEM with n = 7–10 per group. Black dots indicate the individual data points. **(D, E)** Relative mRNA levels of enzymes **(D)** and transcription factors and co-activators **(E)** involved in intracellular redox pathways in the kidney of rats exposed to either the CTRL (grey dots, n = 7–8) or dLAN regime (blue dots, n = 7–10) for 2 and 5 weeks. Data are shown as dot plots with the mean (thick horizontal lines) ± SEM (error bars). CTRL and dLAN groups were compared with the Student’s *t*-test or the Mann–Whitney U test, depending on the normal distribution. *P < 0.05.

Because oxidative stress is an important factor that can influence the recruitment of immune cells into tissues (32), we examined whether dLAN can impact the redox balance in the kidney. Interestingly, rats exposed to dLAN for 2 and 5 weeks showed lower 3-nitrotyrosine (3-NT) protein levels in the renal cortex compared to controls (**Figures 3A, B**). Nitrotyrosine can be formed through nitration of tyrosine residues on proteins and is often measured as a potent indicator of endogenous nitrating agents, especially peroxynitrite, which is known to alter protein structure and function (35). Peroxynitrite is generated by the reaction between nitric oxide (NO) and superoxide anion, and therefore, we analyzed gene levels of enzymes involved in these redox pathways. After 5 weeks, dLAN-exposed rats displayed higher mRNA levels of endothelial NO synthase (*eNos*) in the kidney compared to controls, while renal gene expression of neuronal NO synthase (*nNos*) was not affected by dLAN (**Figure 3D**). No differences between CTRL and dLAN groups were found in anti-oxidative enzymes, such as total SOD activity in the kidney (**Figure 3C**) and renal mRNA levels of *Sod3* and heme oxygenase-1 (*Hmox1*) (**Figure 3D**).

Next, we quantified the gene expression of an important redox-sensitive transcription factor and transcription coactivator in the renal cortex. Following 5 weeks of the dLAN regime, rats displayed higher mRNA levels of nuclear factor erythroid 2-related factor 2 (*Nrf2*) and peroxisome proliferator-activated receptor gamma coactivator-1 alpha (*Pgc-1a*) compared to controls (**Figure 3E**), suggesting a disturbance in the redox balance and up-regulation of the main endogenous antioxidant pathways. We found no effects of dLAN on other transcription factors involved in renal metabolism, peroxisome proliferator-activated receptor alpha (*Pparα*) and peroxisome proliferator-activated receptor gamma (*Pparγ*) (**Figure 3E**).

### dLAN Has No Effects on the Oxidative Burst of Neutrophils in Blood

**Figure 4:**
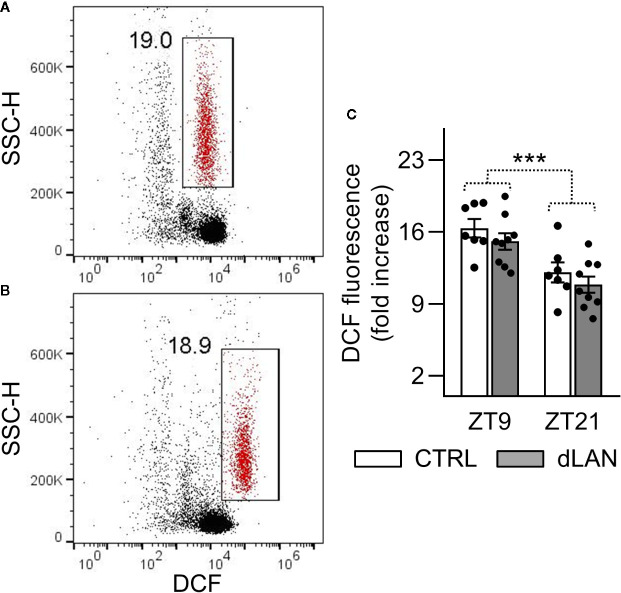
Oxidative burst of peripheral neutrophils was not affected by dLAN. Neutrophils were stimulated by phorbol myristate acetate (PMA) in the whole blood collected at ZT9 and ZT21 from rats exposed to either the control LD regime (CTRL) or dLAN (~2 lx) for 5 weeks. Production of reactive oxygen species (oxidative burst) was analyzed using 2′,7′-dichlorodihydrofluorescein diacetate by flow cytometry. **(A, B)** Representative flow cytometric dot plots showing unstimulated **(A)** and PMA stimulated **(B)** neutrophils (gated population in red). Cell frequencies are displayed next to the gate. **(C)** Quantification of oxidative burst, which was calculated as a fold increase in the median dichlorofluorescein (DCF) fluorescence of the PMA stimulated over the unstimulated neutrophils. Data represent the mean ± SEM. Black dots indicate the individual data points. Two-way repeated ANOVA was used for analysis. ***P < 0.001 indicates significant difference between ZT9 and ZT21.

Oxidative burst in immune cells is an important mechanism that leads to the generation of reactive oxygen and nitrogen species (28). Therefore, we tested the capacity of the circulating neutrophils to induce oxidative burst in response to PMA stimulation in rats exposed to dLAN for 5 weeks. No differences between the dLAN and CTRL rats were detected in the oxidative burst of blood neutrophils (**Figures 4A–C**), indicating that NADPH oxidase, the enzyme responsible for the production of superoxide, was not affected by dLAN in blood neutrophils. Next, we recorded significant daily variation in the oxidative burst of neutrophils, with lower levels at ZT21 compared to ZT9, and this variation remained unaffected in the dLAN regime (**Figure 4C**).

### dLAN Alters Clock Gene Expression in the Kidney

**Figure 5:**
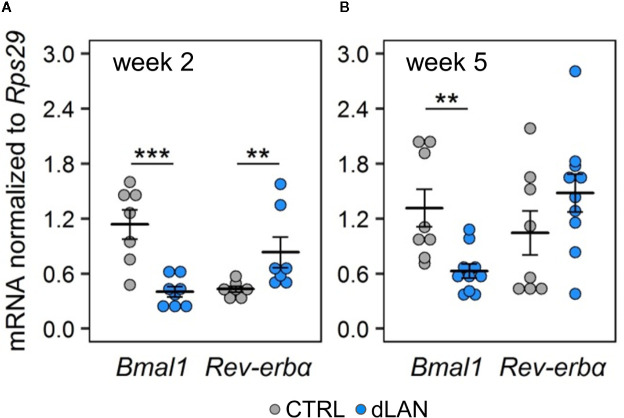
Dim light at night (dLAN) alters clock gene expression in the kidney. **(A, B)** Relative mRNA levels of *Bmal1* and *Rev-erbα* in the renal cortex collected between ZT3 and ZT6 from rats exposed to either the control LD regime (grey dots, n = 7–8) or dLAN (blue dots, n = 7–10) for 2 **(A)** and 5 **(B)** weeks. Data are shown as dot plots with the mean (thick horizontal lines) ± SEM (error bars). Control and dLAN groups were compared with the Student’s *t*-test or the Mann–Whitney U test, depending on the normal distribution. **P < 0.01, ***P < 0.001.

In order to explore the link between dLAN and the circadian clock in the kidney, we analyzed molecular clock components (gene expression of *Bmal1* and *Rev-erbα*) in the renal cortex of rats exposed to dLAN for 2 and 5 weeks. *Bmal1* and *Rev-*erbα represent core clock components of two interconnected feedback loops, which are able to generate and regulate circadian periodicity (3). Moreover, both BMAL1 and REV-ERBα are directly involved in transcriptional control of prominent immune and redox regulators, including *Ccl2* (20, 36) and *Nrf2* (37). Following 2 and 5 weeks of the dLAN regime, the rats showed lower *Bmal1* mRNA levels compared to controls (**Figures 5A, B**) and, simultaneously, 2 weeks of dLAN resulted in increased *Rev-erbα* mRNA levels in the kidney (**Figure 5A**). These results show that dLAN altered gene expression of important components of the molecular clockwork, implying changes in daily oscillations of renal genes that are under direct circadian control.

### dLAN Increases Plasma Corticosterone Levels

**Figure 6:**
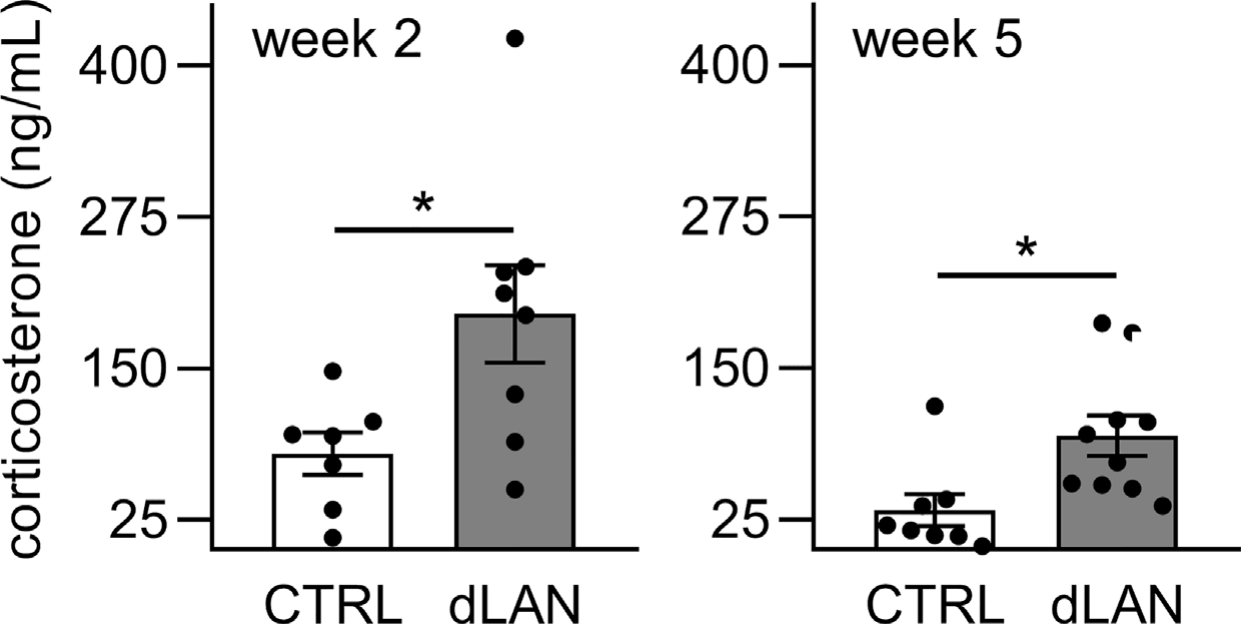
Dim light at night (dLAN) increases plasma corticosterone levels. Rats were exposed to either the control light–dark (LD) regime (CTRL) or dLAN (~2 lx) for 2 and 5 weeks. Blood was collected between ZT3 and ZT6. Data represent the mean ± SEM with n = 7–10 per group. Black dots indicate the individual data points. CTRL and dLAN groups were compared with the Student’s *t*-test. *P < 0.05.

Next, we measured plasma corticosterone concentrations because glucocorticoids are important systemic humoral signals that represent not only potent synchronizers of the peripheral circadian clocks (38) but also regulators of immune cell trafficking into tissues in both homeostatic and inflammatory states (39). We found that rats exposed to dLAN for 2 and 5 weeks showed higher plasma corticosterone levels compared to controls (**Figure 6**), suggesting that glucocorticoid signaling is disturbed under dLAN conditions.

## Discussion

Chronic disruption of the natural LD cycle by dLAN is a pervasive worldwide phenomenon, which has been associated with increased health risks, but the specific mechanisms behind this relation are little understood. In our experiments, we used the model of dLAN (~2 lx), which falls into illuminance levels perceived during night-time from different outdoor or indoor light sources (10, 40). These low levels of nocturnal light have been reported to attenuate the circadian rhythms of locomotor activity and cardiovascular parameters, but the rhythmicity remains preserved (14, 41). In the current study, we showed that chronic exposure to dLAN impaired the daily variation in the main leukocyte subsets in the blood, altered renal expression of *Cd68* and monocyte-attracting chemokine *Ccl2* in association with disturbed renal redox and immune homeostasis.

Daily oscillations in leukocyte numbers in the blood reflect rhythmic egress of mature immune cells from the bone marrow and subsequent migration into peripheral tissues (42). Disturbed photic information by acute experimental jet lag has been shown to abolish the rhythms in leukocyte recruitment to skeletal muscle in mice, indicating the importance of photic entrainment for rhythms in leukocyte trafficking (33). Light at night represents another form of the deregulated LD cycle, and our data demonstrate a disturbed daily variation for the main circulating WBC types, except for neutrophils, in rats after 5 weeks of the dLAN regime, indicating that even very low levels of nocturnal light are sufficient to alter daily rhythms in leukocyte trafficking. In a previous study with mice, the loss in daily variation in classical Ly6C^hi^ monocyte subsets reduced survival and exacerbated inflammatory cytokine production in *Listeria monocytogenes* infection (20). Therefore, we would expect that dLAN through an altered daily variation in blood leukocyte numbers perturbs the anticipatory capability of the host defense mechanisms, which, in turn, increases health risks. Indeed, previous studies have reported that dLAN can alter inflammatory responses, both in the periphery and central nervous system (43, 44).

Rats exposed to dLAN for 2 weeks displayed lower numbers of circulating monocytes compared to controls. Overall peripheral monocyte numbers depend on the release of monocytes from the bone marrow and their recruitment into tissues (20). Thus, we determined different markers of leukocyte recruitment into the kidney since renal infiltration plays an important role in the pathogenesis of many diseases (32, 34), and dLAN has been associated with local activation of inflammatory processes (45). In the current study, 2 weeks of dLAN caused a renal increase of *Cd68* expression, a lysosome-associated macrophage receptor, but CD68 protein levels and the number of CD68-positive cells in the renal cortex were not changed. On the other hand, *Ccl2* expression was reduced in rats after 2 weeks of dLAN. The expression of this potent *monocyte chemokine* is inducible in a variety of cells upon exposure to various inflammatory stimuli (34). Moreover, experimental studies have shown that *Ccl2* expression is under the control of the autonomous molecular clock machinery, and robust daily oscillations in *Ccl2* have been confirmed in endothelial cells of peripheral tissues (33) and peritoneal macrophages, even without immune stimulation (46). Therefore, we can hypothesize that the altered macrophage/monocyte-attracting chemokine relationship caused by exposure to dLAN can be linked to disturbed circadian rhythms in the kidney.

Cellular molecular clocks consist of several interconnected transcriptional–translational feedback loops, which are able to function autonomously and drive rhythmic oscillations across the 24 h cycle (2). In our study, we determined the renal expression of *Bmal1* and *Rev-erbα* because they are the key clock components of two main feedback loops. Clock proteins BMAL1 and CLOCK form a heterodimer, which drives the rhythmic transcription of E-box-containing genes, including *Rev-erbα*, whereas REV-ERBα is a repressive regulator of *Bmal1* expression. Both genes show distinct rhythmicity in the kidney and other peripheral tissues (47). We found altered renal *Bmal1* expression after 2 and 5 weeks of dLAN exposure and altered *Rev-erbα* expression after 2 weeks of the dLAN regime, indicating disruption of the molecular clocks in the kidney. Given the previously published data on the effects of dLAN on clock gene expression in the liver and adipose tissue (17), we would expect that clock genes in the kidney preserve their rhythmicity but the rhythms are attenuated or phase shifted. Nevertheless, further experiments should be designed to evaluate more details. Importantly, both REV-ERBα and BMAL1 are directly involved in the rhythmic transcriptional control of *Ccl2* expression and, in this way can coordinate the daily variation in monocyte dynamics between the blood and tissues (20, 36). In line with these data, our results suggest that dLAN-induced disruption of molecular clock machinery in the kidney can attenuate the circadian control of leukocyte trafficking through deregulation of chemotactic factors.

Circadian clocks exert essential control over cellular redox homeostasis (48), and prior studies have shown that disruption of molecular clocks can induce oxidative stress and cause oxidative damage in various body tissues (49, 50). Moreover, increased intrarenal oxidative stress has been associated with immune cell recruitment, and these processes can function in a self-amplifying cycle (32). Immune cells, especially neutrophils and macrophages, produce reactive oxygen and nitrogen species, such as the superoxide anion and NO, which react to form peroxynitrite (35). Interestingly, we found reduced 3-NT protein levels, a typical biomarker of peroxynitrite, in the renal cortex following 2 and 5 weeks of dLAN. This decrease could be explained by changed availability of superoxide or NO for peroxynitrite formation. Our data showed up-regulated renal *eNos* in rats exposed to dLAN for 5 weeks, suggesting that NO production was not reduced but NO can be consumed by NO dependent signaling pathways in the cell (51). For example, in parallel, we found up-regulation of renal *Nrf2* mRNA in dLAN exposed rats and there is evidence that NO can stimulate both NRF2 activation and *Nrf2* gene expression (52). On the other hand, we detected no changes caused by dLAN in renal enzymatic systems controlling superoxide levels and no changes in the oxidative burst of peripheral neutrophils. Nevertheless, the dLAN-induced limited availability of peroxynitrite can indicate not only redox imbalance in the kidney but it can also interfere with the function of chemokines, including CCL2, because nitration of CCL2 has been found to change an ability of this chemokine to stimulate monocyte migration (53).

Following 5 weeks of the dLAN regime, we found increased *Nrf2* and *Pgc-1a* expression in the renal cortex, which further indicated the disturbed redox homeostasis in the kidney. NRF2 is a key cytoprotective transcription factor that activates the expression of a number of cytoprotective genes involved in antioxidant and detoxifying responses (54). Simultaneously, NRF2 signaling is implicated in the suppression of the proinflammatory pathways (55). Many of the effects of NRF2 are shared by PGC-1α, a tissue-specific transcription coactivator that coordinates specific biological programs, including antioxidant and anti-inflammatory responses, *via* interactions with different transcription factors (56). At the transcriptional level, several pathways associated with oxidative, energy or immune stress have been identified to up-regulate *Nrf2* and *Pgc-1a* in different cell types (56). Given that our data did not directly confirm oxidative stress or inflammation in the kidney of dLAN-exposed rats, it is likely that increased *Nrf2* and *Pgc-1a* expression can be associated with disturbed molecular circadian clocks in the kidney. Direct circadian regulation has been indicated for both NRF2 and PGC-1α (37, 57). The BMAL1/CLOCK heterodimer controls the rhythmic transcriptional activation of *Nrf2* in the murine lungs, and a disruption of the circadian clock in Clock^Δ19^ mutant mice resulted in a loss of rhythmicity in the NRF2-mediated antioxidant defense, which is linked to elevated oxidative damage and pulmonary fibrosis (37). Therefore, we can hypothesize that dLAN-induced circadian clock deregulation in the kidney can compromise the time of day dependence of cytoprotective defense mechanisms, which, in turn, can lead to adverse health consequences, especially in combination with dLAN and other environmental or health challenges (58). However, future studies are required to better understand this potential link.

The time-of-day dependent mobilization of mature leukocytes from the bone marrow into the circulation and subsequent migration into tissues is centrally orchestrated by the SCN *via* autonomic nervous and endocrine pathways (5, 42). Therefore, it is adequate to consider that chronic exposure to dLAN impairs temporal organization of immune cell trafficking by compromising these central clock-output pathways. Indeed, we recently showed that rats exposed to dim nocturnal light displayed diminished 24 h variability in blood pressure and heart rate and enhanced cardiovascular response to norepinephrine, suggesting changes in adrenergic signaling under the dLAN conditions (14). Another important central clock-derived output is melatonin, which is produced in the pineal gland and serves as a sensitive marker of non-image forming responses to light (25). A recent review on melatonin under dLAN conditions revealed that nocturnal melatonin can be suppressed by surprisingly low light intensities in the most studied vertebrate groups, including humans (40). In line with these data, we demonstrated that nocturnal light exposure at the level of 2 lx resulted in suppression of plasma melatonin concentrations during the night phase of rats after both 2 and 5 weeks of the dLAN regime (14). The melatonin peak during darkness signals homing and increases the repopulation potential of the hematopoietic stem and progenitor cells within the bone marrow (59), indicating that suppression of melatonin rhythm might play a role in dLAN-induced impaired leukocyte trafficking.

In addition to melatonin, glucocorticoids are other key endocrine signals that couple the central and peripheral circadian clocks (60), and are important modulators of different immune processes (61). Corticosterone, a dominant glucocorticoid in rats, is released from the adrenal cortex in response to the adrenocorticotropic hormone in a circadian manner (38). Corticosterone levels increase during stress and during the circadian-regulated peak at the beginning of the active phase (26). While deregulated LD cycles by exposure to phase shifts (26) or constant light eliminated corticosterone daily rhythms in rats (62), under low intensities of nocturnal light plasma corticosterone remains rhythmic, but may be attenuated or phase shifted, resulting in increased daytime levels (15). These effects are also supported by our results, which showed that 2 and 5 weeks of dLAN increased the daytime plasma corticosterone concentrations compared to the control regime. Importantly, glucocorticoid signaling is involved in the regulation of daily changes in T cell redistribution between the blood and lymphoid tissues under homeostatic conditions (63), and, thus, alterations in corticosterone production during dLAN exposure may represent an additional pathway, which contributes to deregulation of daily variation in leukocyte trafficking.

Based on our results, we cannot distinguish whether changes in daily variation of circulating leukocytes and impaired renal immune and redox homeostasis induced by dLAN reflect suppressed or phase shifted rhythms of leukocytes indicating a need of future experiments to analyze the whole 24-h rhythms under dLAN conditions. Moreover, in our current study we addressed dLAN effects in male rats but sex specific differences in the sensitivity to this environmental factor may exist and should be studied also in females.

In summary, our results revealed that chronic exposure to nocturnal low-intensity light has a clear impact on the immune parameters, even under the steady state. We found impaired daily variation in the main leukocyte subsets in the blood and altered renal expression of *Cd68* and monocyte-attracting chemokine *Ccl2*, which was associated with a disturbed redox balance and up-regulation of the important protective pathways in the kidney. These dLAN-induced effects are probably mediated by the compromised circadian control, both at the level of renal molecular clocks and endocrine central clock output pathways. Taken together, we assume that dLAN-disturbed immune balance may represent a potential circadian-dependent immunomechanism, which can interfere with predictive capability of immune defense processes to cope with other environmental challenges.

## Data Availability Statement

The datasets generated for this study are available on request to the corresponding author.

## Ethics Statement

The experimental procedure was approved by the Ethical Committee for the Care and Use of Laboratory Animals at the Comenius University in Bratislava, Slovak Republic, and the State Veterinary Authority of the Slovak Republic.

## Author Contributions

MO, NM, VR, and MZ performed the experiments. MO collected and analyzed the data and wrote the manuscript. NM collected data from flow cytometry and renal immunofluorescence. MM performed Western blotting and SOD activity analyses. MZ and MO conceived and supervised the study. All authors contributed to the article and approved the submitted version.

## Funding

The study was supported by the Slovak Research and Development Agency APVV-17-0178 and the Scientific Grant Agency of the Ministry of Education of the Slovak Republic VEGA 1/0492/19.

## Conflict of Interest

The authors declare that the research was conducted in the absence of any commercial or financial relationships that could be construed as a potential conflict of interest.
