# Supplementary material for: Dim Light at Night Impairs Daily Variation of Circulating Immune Cells and Renal Immune Homeostasis
Source: Front Immunol. 2021 Jan 22;11:614960. doi: 10.3389/fimmu.2020.614960 (PMC7862740; doi:10.3389/fimmu.2020.614960)
Supplement: Supplementary file 1 [file DataSheet_1.docx]

Supplementary Material

## Supplementary Figures


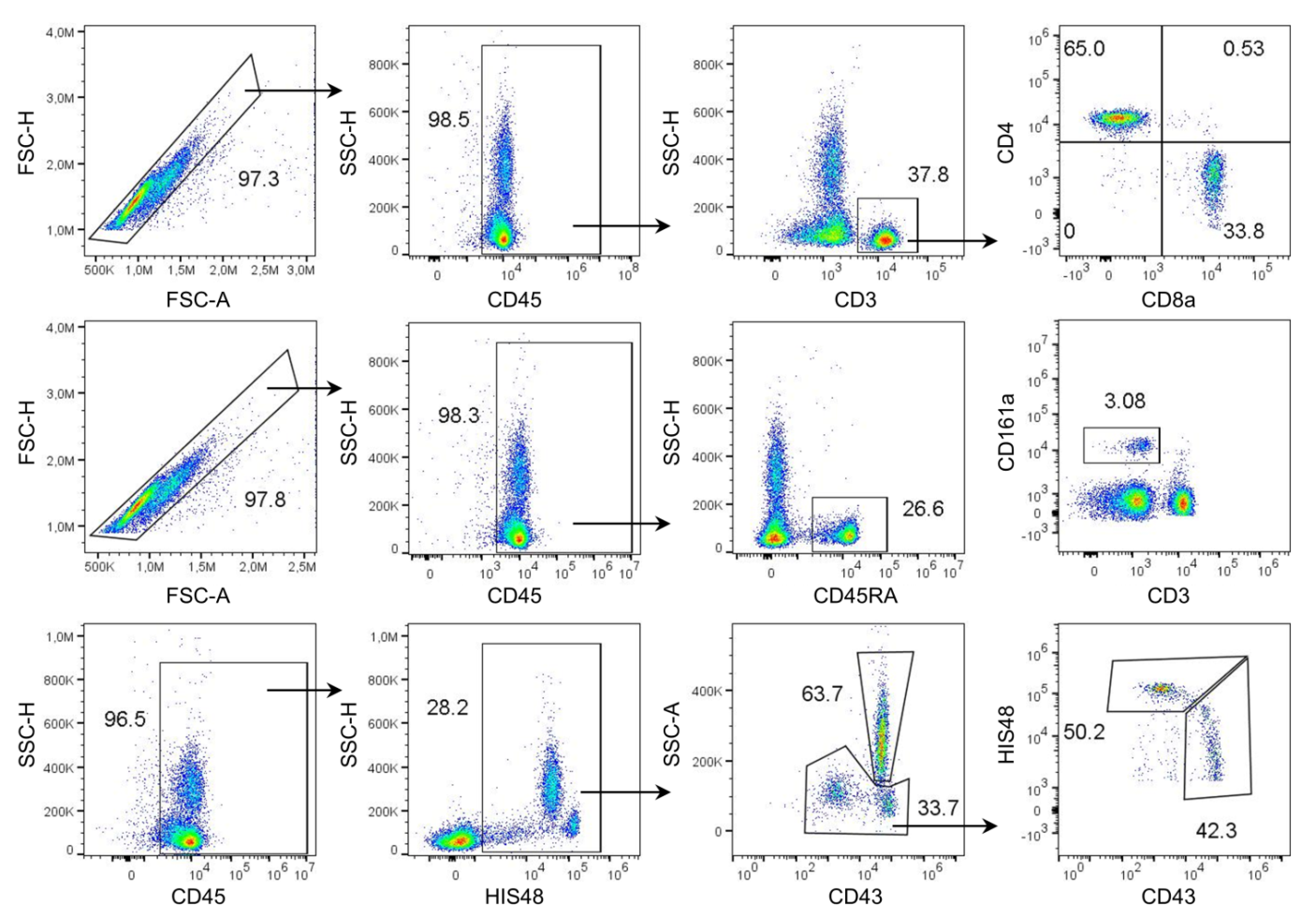


**FIGURE S1.** The gating strategy for identifying individual populations of peripheral blood leukocytes in rats. Isolated cells were stained using 3 separate panels of antibodies. In all cases, cells were gated on single cells and further on CD45^+^ cells (total leukocytes). Panel 1 (upper row) was used to discriminate CD3^+^ T cells that were next gated into CD8^+^ cytotoxic and CD4^+^ helper T cells. Panel 2 (middle row) was used to discriminate CD45RA^+^ B cells and CD3^-^CD161a^+^ NK cells. Panel 3 (lower row) was first used to discriminate HIS48^+^ cells, which were subsequently gated into neutrophils by higher granularity and monocyte subsets by reciprocal expression of CD43 and HIS48 markers: CD43^lo^HIS48^hi^ classical and CD43^hi^HIS48^lo^ non-classical monocytes. Cell frequencies are shown next to the each gate.

## Supplementary Tables

**TABLE S1.** Primer sequences for real-time PCR.

| **Gene** | **Accession Number** | **Forward primer** | **Reverse primer** |
| --- | --- | --- | --- |
| ***Cd3d*** | NM_013169.1 | 5’-CTCATCTTGGGCAAAGGCATC-3’ | 5’-ACAGTTCTGGCACATTCGGTAA-3’ |
| ***Cd68*** | NM_001031638.1 | 5’-CGCATCTTGTACCTGACCCA-3’ | 5’-GCTCTGATGTCGGTCCTGTTT-3’ |
| ***Tlr4*** | NM_019178.1 | 5’-TCACAACTTCAGTGGCTGGAT-3’ | 5’-TTGTCTCCACAGCCACCAGAT-3’ |
| ***Ccr2*** | NM_021866.1 | 5’-GCCACCACACCGTATGACTATG-3’ | 5’-TCCAAGCTCCAATTTGCTTCAC-3’ |
| ***Cd36*** | NM_031561 | 5’-CGGCGATGAGAAAGCAGAAA-3’ | 5’-GGCTCATCCACTACTTATTTTCC-3’ |
| ***Ccl2*** | NM_031530.1 | 5’-TGTCTCAGCCAGATGCAGTTAAT-3’ | 5’-AGTTCTCCAGCCGACTCATTG-3’ |
| ***Ccl5*** | NM_031116.3 | 5’-ACCTTGCAGTCGTCTTTGTC-3’ | 5’-TCTTGAACCCACTTCTTCTCTGG-3’ |
| ***Icam1*** | NM_012967.1 | 5’-ACGGAGCCAATTTCTCATGC-3’ | 5’-TCAGGACCCTAGTCGGAAGA-3’ |
| ***Vcam1*** | NM_012889.1 | 5’-GGAGTGAATCTGGTTGGGAGA-3’ | 5’-CAGCACATGTCAGAACAACGG-3’ |
| ***Bmal1*** | NM_024362.2 | 5’-CACCTTGCGGAATGTCACAG-3’ | 5’-TACTTCCTTGGTCCACGGGT-3’ |
| ***Rev-erbα*** | NM_145775.2 | 5’-TCCCACATACTTCCCACCATCA-3’ | 5’-CACTCGGCTGCTGTCTTCCAT-3’ |
| ***Pparα*** | NM_013196.1 | 5’-GACTAGCAACAATCCGCCTT-3’ | 5’-GAAGAATCGGACCTCTGCCT-3’ |
| ***Pparγ*** | NM_001145367.1 | 5’-TCCAAGAATACCAAAGTGCGA-3’ | 5’-CCATGAGGGAGTTTGAAGGC-3’ |
| ***Pgc-1α*** | NM_031347.1 | 5’-AACGATGACCCTCCTCACAC-3’ | 5’-GTTGTTGGTTTGGCTTGAGCA-3’ |
| ***Nrf2*** | NM_031789.2 | 5’-GCTGCCATTAGTCAGTCGCT-3’ | 5’-CGGTGGGTCTCCGTAAATGG-3’ |
| ***Hmox1*** | NM_012580.2 | 5’-TCAGAAGGGTCAGGTGTCCAG-3’ | 5’-GCAGCTCCTCAGGGAAGTAGA-3’ |
| ***Nos1*** | NM_052799.1 | 5’-AATTGGCAGAGGCCGTCAAG-3’ | 5’-TGTGGCGTAGAGAATGGTCG-3’ |
| ***Nos3*** | NM_021838.2 | 5’-TAACTCGATCAAAAGGAGTGGT-3’ | 5’-GCAGTCCCGAGCATCAAAT-3’ |
| ***Sod3*** | NM_012880.1 | 5’-GCAGAACACCTCCAACCACG-3’ | 5’-CCACGAAGTTGCCGAAGTCC-3’ |
| ***Rps29*** | NM_012876.1 | 5’-GCTGAACATGTGCCGACAGT-3’ | 5’-GGTCGCTTAGTCCAACTTAATGAA-3’ |

Abbreviations: *Bmal1*, brain and muscle Arnt-like protein-1; *Ccl2*, C-C motif chemokine ligand 2; *Ccl5*, C-C motif chemokine ligand 5; *Ccr2*, C-C chemokine receptor type 2; *Hmox1*, heme oxygenase-1; *Icam1*, intercellular cell adhesion molecule-1; *nNos*/*Nos1*, neuronal nitric oxide synthase; *eNos*/*Nos3*, endothelial nitric oxide synthase; *Nrf2*, nuclear factor erythroid 2-related factor 2; *Pgc*-*1α*, peroxisome proliferator-activated receptor gamma coactivator-1 alpha; *Pparα*, peroxisome proliferator-activated receptor alpha; *Pparγ*, peroxisome proliferator-activated receptor gamma; *Rps29*, ribosomal protein S29; *Sod3*, superoxide dismutase-3; *Tlr4*, toll-like receptor 4; *Vcam1*, vascular cell adhesion molecule-1
